# Classification and Characterization of the Manoor Valley’s (Lesser Himalaya) Vegetation from the Subtropical-Temperate Ecotonal Forests to the Alpine Pastures along Ecological Variables

**DOI:** 10.3390/plants11010087

**Published:** 2021-12-28

**Authors:** Inayat Ur Rahman, Aftab Afzal, Zafar Iqbal, Mashail Nasser Alzain, Al-Bandari Fahad Al-Arjani, Abdulaziz A. Alqarawi, Elsayed Fathi Abd_Allah, Niaz Ali, Shazia Sakhi, Muhammad Azhar Khan, Uzma Khan, Farhana Ijaz, Samina Mumtaz, Eduardo Soares Calixto

**Affiliations:** 1 Department of Botany, Hazara University, Mansehra 21300, Pakistan; zafar.iqbal@hu.edu.pk (Z.I.); niazalitk25@gmail.com (N.A.); azharfinal@gmail.com (M.A.K.); uzmaqau2003@yahoo.com (U.K.); 2 William L. Brown Center, Missouri Botanical Garden, 4344 Shaw Blvd, St. Louis, MO 63110, USA; 3 Department of Biology, College of Sciences, Princess Nourah Bint Abdulrahman University, Riyadh 11451, Saudi Arabia; mnAlZain@pnu.edu.sa; 4 Botany and Microbiology Department, College of Science, King Saud University, Riyadh 11451, Saudi Arabia; aalarjani@ksu.edu.sa; 5 Department of Plant Production, College of Food and Agriculture Science, King Saud University, Riyadh 11451, Saudi Arabia; alqarawi@ksu.edu.sa (A.A.A.); eabdallah@ksu.edu.sa (E.F.A.); 6 Center of Plant Sciences and Biodiversity, University of Swat, Swat 19200, Pakistan; shaziasakhi@gmail.com; 7 Department of Animal Sciences, Karakoram International University, Gilgit-Baltistan 15100, Pakistan; samina@kiu.edu.pk; 8 Department of Biology, University of Missouri, St. Louis, MO 63166, USA; calixtos.edu@gmail.com; 9 Entomology and Nematology Department, University of Florida, Gainesville, FL 32611, USA

**Keywords:** vegetation structure, environmental variables, PC-ORD, plant community assembly, Himalaya

## Abstract

Plant species are distributed in different types of habitats, forming different communities driven by different sets of environmental variables. Here, we assessed potential plant communities along an altitudinal gradient and their associations with different environmental drivers in the unexplored Manoor Valley (Lesser Himalaya), Pakistan. We have implemented various ecological techniques and evaluated phytosociological attributes in three randomly selected 50 m-transects within each stand (a total of 133) during different seasons for four years (2015–2018). This phytosociological exploration reported 354 plant species representing 93 different families. The results revealed that the Therophytic life form class dominated the flora, whereas Nanophyll dominated the leaf size spectra. There were a total of twelve plant communities identified, ranging from the lowest elevations to the alpine meadows and cold deserts. The maximum number of species were found in *Cedrus–Pinus–Parrotiopsis* community (197 species), in the middle altitudinal ranges (2292–3168 m). Our results showed that at high altitudes, species richness was reduced, whereas an increase in soil nutrients was linked to progression in vegetation indicators. We also found different clusters of species with similar habitats. Our study clearly shows how altitudinal variables can cluster different plant communities according to different microclimates. Studies such as ours are paramount to better understanding how environmental factors influence ecological and evolutionary aspects.

## 1. Introduction

The study of vegetation classification based on species co-occurrence [1,2] and its relationship to ecological variables [3] is known as phytosociology. This field has specified major strategies and methodologies that may be linked to vegetation mapping [4,5], and biodiversity conservation [6]. Plant biodiversity research generally focuses on species diversity [7,8,9] and species-level measurement [10]. It is greatly influenced by a variety of environmental variables [11,12,13], such as climatic, edaphic, and geographic variables [13,14,15,16,17,18]. Plant associations/communities with a well-defined structure in respect to ecological variables can be described physiognomically as well as floristically [19,20,21]. Floristic diversity and biological spectra rely on topography as well as other environmental variables. For instance, biological spectrum mirrors the existing ecological and natural surroundings [22,23]. They are the plant characteristics that have been widely applied in vegetation research [24]. High mountains are major hotspots for endemics across the world [25,26,27,28].

It is well understood that altitude is a complex factor along which many environmental gradients [29] and species diversity [15,30] change accordingly. Biological as well as the environmental gradients interact to govern the distribution of species richness all around the altitudinal gradient [31,32,33]. The species richness of higher plant species has been reported to have increased in temperate latitudes [34]. The relationship between vegetation and ecological diversity is reflected as a percentage of the ecosystem’s overall quality [35]. The altitudinal gradient has a greater influence on temperature in mountainous regions than latitude, and the rate of decrease is considerably faster in summer than in winter, resulting in altitudinal vegetation zonation [29].

Plant species are found in a diverse range of environments, forming different communities driven by different sets of environmental variables [6,36,37]. Many ecological experts have recognized distinct types of forests in Pakistan [38,39,40,41], but it is not clear how and which environmental variables drive plant diversity and community structure in most alpine ecosystems. In this context, we used different multivariate approaches to assess potential plant communities along an altitudinal gradient and their associations with different environmental drivers, namely climatic, edaphic, and physiographic variables. In sum, we assessed (i) which potential plant communities are present in the subtropical-temperate ecotonal forests to the alpine pastures; (ii) which plant species are most representative each plant community; (iii) which environmental variables most determine plant community structure in this region; and (iv) which species are distribution in each community based on their biological spectrum (life form and leaf size). Since there is a significant variability in environmental gradients with respect to the altitude and different plant species are adapted to a set of micro-climatic conditions [42,43,44], we hypothesized that there would be different plant communities along the elevational gradient, with specific plants related to particular sets of environmental variables. Importantly, statistical methodologies in vegetation ecology, such as multivariate analysis [45], have evolved in recent decades, allowing researchers to evaluate the impact of ecological variables on large groups of plants [6,43,46,47,48]. Therefore, we took advantage of these advanced statistical approaches to assess viable low-dimensional summaries of field information by advantageous and objective means [49].

## 2. Materials and Methods

### 2.1. Study Area

**Figure 1:**
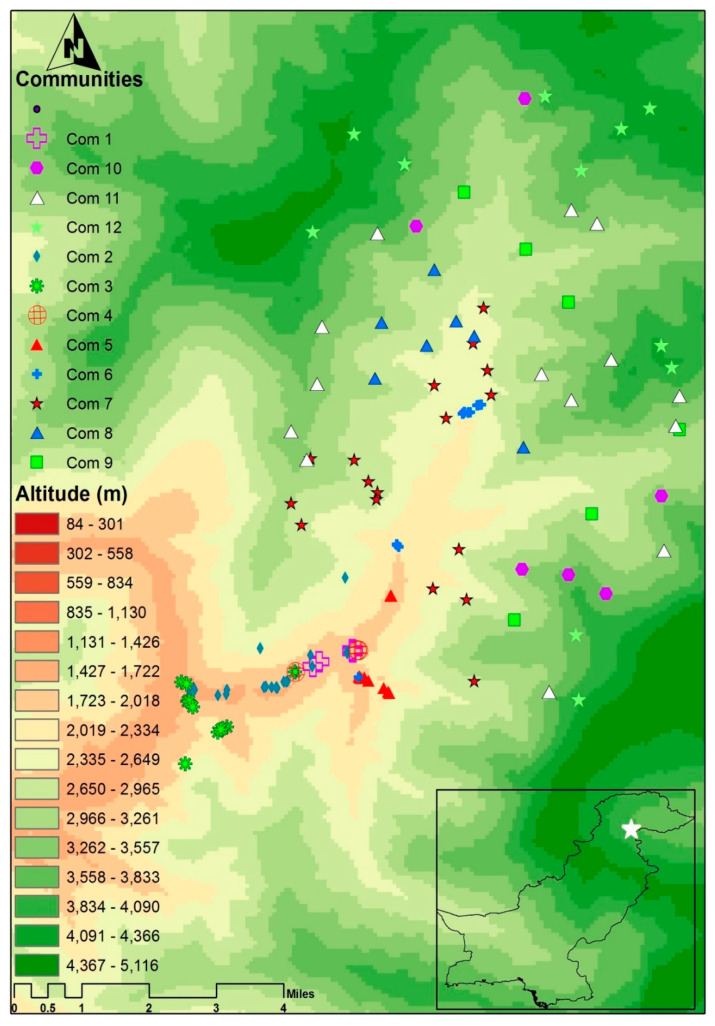
GIS map (generated using ArcGIS version 10.1) depicting the altitudinal layers and distribution pattern of communities of the studied area (Manoor Valley). Com 1: *Salix–Sorbaria–Impatiens*, Com 2: *Indigofera–Juglans–Isodon*, Com 3: *Cedrus–Cynodon–Isodon*, Com 4: *Indigofera–Parrotiopsis–Bistorta*, Com 5: *Sambucus–Cedrus–Desmodium*, Com 6: *Indigofera–Cedrus–Pinus*, Com 7: *Cedrus–Pinus–Parrotiopsis*, Com 8: *Pinus–Viburnum–Cedrus*, Com 9: *Abies–Picea–Juniperus*, Com 10: *Juniperus–Sibbaldia–Juniperus*, Com 11: *Sibbaldia–Bergenia–Rheum*, Com 12: *Poa–Bistorta–Primula*.

Geographically, the Manoor Valley [4,50,51] is situated in the north-western part of Pakistan (34.68165 N to 34.83869 N latitude and 73.57520 E to 73.73182 E longitude; 1580 to 4677 m elevation above sea level) and is part of the Himalayan mountain range. The multiple elevational layers of the research area are depicted on a georeferenced map (Figure 1). The wide gap in in elevation demonstrates that the climate differs from lower altitudinal ranges [4] to the alpine meadows [42]. The study area is located on the Indian Plate’s north-western boundary [52], which has immense phytogeographic and floristic significance.

### 2.2. Vegetation Sampling and Herbarium work

The vegetation of the study area (Manoor Valley) was surveyed and quantified [53] during four consecutive years, from 2015 to 2018, along the environmental variables [43]. The line transect method was adopted for vegetation sampling [54,55,56,57,58]. The study area was subdivided into 133 stands (sampling plots). Each stand was replicated thrice (three transects of 50 m in each stand) [53,59]. The interval between each transect was 100 m and the interval between the stands was 200 m. The phytosociological attributes (i.e., density, frequency and their relative values, and importance value (IV)) were employed on the recorded data of each stand [4,60,61]. The species were further ranked with the highest IV and considered the representative species [19,62]. Similarly, plant communities were designated based on three dominant species [63,64,65,66]. Moreover, both attributes of the biological spectrum (life form and leaf size spectra) were recognized by the following [67]. Methods for collecting specimens, their labelling, pressing, drying, poisoning, and mounting were adopted by the following [68,69]. Their identification was achieved with the aid of Flora of Pakistan [70,71,72] and submitted to the Herbarium of Hazara University, Mansehra (Pakistan).

### 2.3. Ecological Variables

The slope angle, aspect and exposure were recorded at each stand using a clinometer, while the altitude, longitude, and latitude were recorded by the Global Positioning System (GPS). Two hundred grams of soil samples from three randomly selected transects within each sampling stand (0–30 cm depth) were collected [73] and mixed thoroughly to make a composite sample [74], stored in a sterile polythene bag and labeled. All the samples were submitted to the Soil and Water Testing Laboratory at the Model Farm Service Center in Mansehra, Pakistan, for analysis of various physicochemical parameters such as soil pH [75], and texture (loam, clay, silt and sand) [76], organic matter (OM%) [77], nitrogen (N) [78], potassium (K), phosphorous (P) [79], calcium carbonate (CaCO_3_) [80,81,82], and electric conductivity (EC) [76]. Moreover, other climatic variables were measured by a small remote weather station (Kestrel 4000 weather and environmental tracker) like temperature, humidity, wind speed (WS), barometric pressure (BP), wet bulb (WB), heat index (HI), and dew point (DP) to record the data at each transect and then average values were calculated at stand level [43].

### 2.4. Statistical Analyses

Multivariate analysis was carried out to analyze the recorded data of species and ecological variables resulting from the field observations [49,83] to find out the relationship among them [84,85]. The recorded species and sampled stands were constrained in association to the ecological variables [86,87], which were divided into geographic, slope aspect, edaphic, and climatic variables. For the identification and classification of plant communities [53], the two-way indicator species analysis (TWINSPAN) was processed using PC-ORD version 5.0 [87,88,89]. A georeferenced map was generated with ArcGIS version 10.1 to depict the distribution pattern of plant communities.

Canonical correspondence analysis (CCA) was used to ordinate species and samples along the ecological variables [90,91] using CANOCO version 5 [92,93], and we performed a variation partitioning test (partial CCA) to evaluate how explanatory attributes (climatic, edaphic, geographic, and slope) drive the plant species distribution. First, we built the best model with the lowest number of variables (those that most explain variance), through the *step* function in R. Next, we also evaluated multicollinearity between variables of the final model using Variance Inflation Factor (VIF), and we removed any variable with VIF >10, one at a time. Non-multidimensional scaling ordination (NMDS) [89,94] was performed using the software R 4.0.1 [95,96,97]. NMDS was conducted to evaluate the correlation of recognized plant communities with their associated species.

## 3. Results

A total of 12 plant communities were recognized, each representing for different indicator species. Each community was associated to a set of variables, but altitude, Slope (ES), Slope (SE), Slope (SW), Slope (WN), electric conductivity (EC) and heat index were the most significant variables driving species distribution in the present study. Therophytes and Hemicryptophytes, and Nanophyll, were the most frequent type of life form and leaf size, respectively, present in the communities found. Below we discuss these results in detail.

### 3.1. TWINSPAN Classification

TWINSPAN, which is based partitioning reciprocal averaging ordination space, was used to classify 354 species and 133 stands. Two large different clusters, which show a high cluster heterogeneity value (Lambda = 0.814). One of these clusters had eight different communities and was formed by 93 sampling sites, while the other cluster presented four communities structured into 40 sites. Furthermore, different subdivisions observed were within these two large groups with cluster heterogeneity values of less than 0.4: a total of 12 major plant communities were recognized, from subtropical-temperate ecotonal forests (1580 m) to the alpine meadows and cold deserts (4278 m) of the Manoor Valley, Lesser Himalayas. Each community was composed of different groups of indicator species recorded at different altitudes (Figure 1).

### 3.2. Vegetation Characterization of Plant Communities

**Figure 2:**
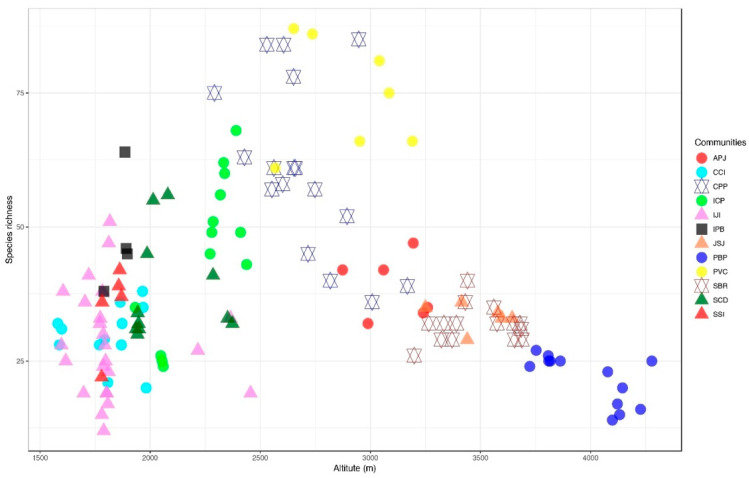
The relationship between the altitude and the species richness of 12 plant communities.

**Figure 3:**
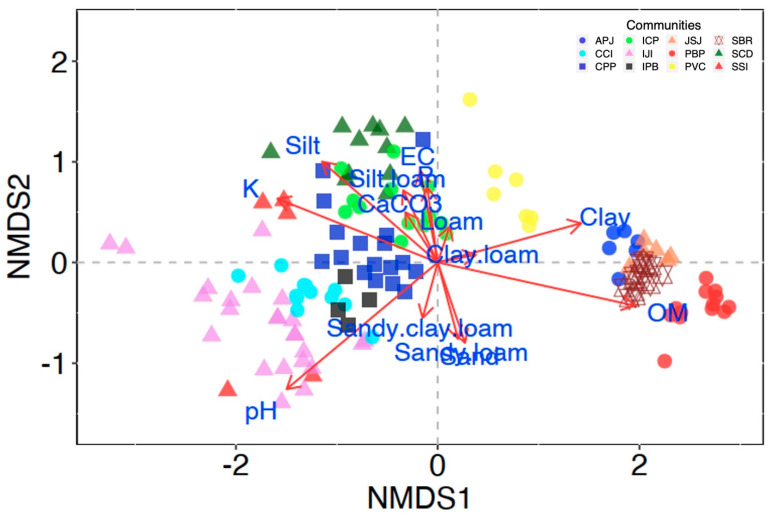
The NMDS ordination reveals the relationship between communities and edaphic variables. The length of the arrows illustrates the influence range, while the direction shows the correlation of the variables with plant communities. Plant communities that are close together or on the same axis have a positive correlation. The codes represent community types.

In total, 12 major plant communities were established by TWINSPAN. All the twelve recognized plant communities were indicated with distinct symbols and colours. The GIS map shows the elevational layer and communities of the study area—illustrating the recognition and distribution of plant communities (Figure 1) along the ecological variables (Figure 2 and Figure 3 and Supplementary data Figures S1–S4).

#### 3.2.1. *Salix–Sorbaria–Impatiens* Community

This community (SSI) was recorded between altitudinal ranges of 1782.3–1869.5 m (5 stands) with 65 associated species. The indicator plant species of the SSI community were *Salix alba*, *Sorbaria tometosa and Impatiens bicolor* with highest IV values of 9.12, 5.51, and 5.29, respectively. Other frequent species were *Clematis grata*, *Bromus secalinus*, *Fragaria nubicola*, *Rumex nepalensis*, *Ficus carica*, *Salvia moorcroftiana*, *Indigofera heterantha*, *Bistorta amplexicaulis*, *Crotalaria* sp., *Filipendula vestita* and *Desmodium elegans.* Rare species with lower IV values included *Withania somnifera, Trachyspermum amii, Clinopodium vulgare*, *Paspalum dilatatun*, *Piptatherum aequiglume*, *Bauhinia variegata* and *Salix tetrasperma*. The life form spectra was dominated by Therophytes (36.92% of species), followed by Hemicryptophytes with 15.38% of species (Table 1). Nanophyll dominated the leaf size spectra with 30.77% of the species, followed by Mesophyll and Microphyll with 27.69% species each (Table 1). The ecological variables that strong and positively influenced the SSI community were pH (6.5–7), temperature (26.1–27.2 °C), HI (26.8–29.1), and BP (808.1–816.1) (Supplementary data Figure S1). Other important variables such as altitude and windspeed (0–1.5 m/s) were found in negative association with SSI community. Nevertheless, the SSI community’s species diversity was restricted by low OM (0.65–1.15%) and P (9.6 mg/kg) (Figure 3 and Supplementary data Figure S2).

#### 3.2.2. *Indigofera–Juglans–Isodon* Community

This plant community (IJI) was recognized in 24 stands at an altitude ranging from 1597 to 2456 m with 113 associated species (Table 1). *Indigofera heterantha*, *Juglans regia*, and *Isodon rugosus* were recognized as the indicator species that dominated the community with IV values of 6.94, 5.00, and 4.37, respectively. Other co-dominant species were *Cynodon dactylon*, *Ziziphus* sp., *Micromeria biflora*, *Leptopus chinensis*, *Rumex hastatus*, *Ailanthus altissima* and *Impatiens bicolor*. Moreover, species that were rarely recorded in this community were *Dactylis glomerata*, *Achyranthes aspera*, *Lamium album*, *Bupleurum longicaule*, *Ricinus communis*, *Malus domestica*, *Pyrus pashia*, *Dodonaea viscosa*, *Filipendula vestita*, *Lactuca tatarica*, *Pinus roxburghii* and *Xanthium strumarium*. Therophytes dominated the biological spectrum with 33.04% of plant species, followed by Hemicryptophytes (22.32%), Chamaephytes and Nanophanerophytes (10.71%) each (Table 1). Nanophyllous class dominated the leaf size spectra, accounting for 35.71% of plant species, followed by microphyll (26.79%), and mesophyll (20.54%). The IJI community was supported by abundant limestone, granite, and sandstone. High temperatures (32.5 °C), pH (5.7–7.1), K (200–235 mg/kg), and HI (26.8–29.1) all had an impact on the indicators of the IJI community and their associated species, which were distributed on a slope angle of 28–75° (Figure 3 and Supplementary data Figures S1–S4).

#### 3.2.3. *Cedrus–Cynodon–Isodon* Community

This community (CCI) was recognized in 12 stands between altitudes of 1580.8–1982 m. *Cedrus deodara* (11.77 IV), *Cynodon dactylon* (10.42 IV) and *Isodon rugosus* (6.02 IV) were recorded as the leading indicators of this plant community. Other co-dominant species were *Dryopteris wallichiana*, *Oxalis corniculate*, *Medicago sativa*, *Cyperus rotundus*, *Fragaria nubicola*, *Adiantum capillus-veneris*, *Impatiens bicolor*, *Trifolium repens*, *Clematis grata*, *Artemisia absinthium*, *Leptodermis virgata*, *Convolvulus arvensis*, *Tagetes minuta*, *Cirsium arvense*, *Persicaria capitata* and *Hedera nepalensis*. Nonetheless, *Silybum marianum*, *Dicliptera bupleuroides*, *Cichorium intybus*, *Pteridium aquilinum*, *Geranium nepalense*, *Conyza japonica*, *Malvastrum coromandelianum*, *Saussurea* sp., *Pinus roxburghii*, *Achyranthes bidentata* and *Pyrus pashia* were found as the rare species with lower IV values. Therophytes dominated the life form spectra with 33.80% of species, followed by Hemicryptophytes with 25.35% of species, Chamaephytes with 12.68% of species, Nanophanerophytes and Geophytes each with 9.86% of species (Table 1). Nanophyll dominated the leaf size spectra with 40% of the species, followed by Microphyll with 30.67% of the species, and Leptophyll and Mesophyll with 10.67% of the species. Atmospheric humidity (53.6–74.9%), DP (15.5–19.1%), temperature (20.9–28.4 °C), EC (0.56–2.24 dsm^−1^), OM (0.6–1.25%) and pH (5.9–7.3), were the most influential ecological variables that influenced the composition as well as distribution of plant species of CCI community. Aspect (N-E), slope angle (24–50°), and silty loam soil texture were also important significant variables of the CCI community (Supplementary data Figures S1–S4).

#### 3.2.4. *Indigofera–Parrotiopsis–Bistorta* Community

The IPB community was recognized on the aspect (W-N) with a slope angle (45–80°) in four stands at an altitudinal range of 1789.6–1896.3 m, which has a total of 87 species associated species (Table 1). *Indigofera heterantha*, *Parrotiopsis jacquemontii*, *Bistorta amplexicaule* were recorded as the dominant species in this community with highest IV values of 12.34, 9.05 and 6.18, respectively. Other characteristic species based IV were *Indigofera hebepetala*, *Clematis grata*, *Bromus secalinus*, *Cynodon dactylon*, *Clinopodium vulgare*, *Cynoglossum glochidiatum*, *Urochloa panicoides*, *Isodon rugosus*, *Pimpinella stewartia*, *Berberis lycium*, *Dysphania ambrosioides*, *Pyrus pashia* and *Poa infirma*. Rare species of this community with minimum IV values were *Leptodermis virgata*, *Cynoglossum apenninum*, *Commelina benghalensis*, *Cannabis sativa*, *Bergenia ciliata*, *Crotalaria* sp., *Malva neglecta*, *Ficus carica*, *Salix alba*, *Achyranthes bidentata*, *Malvastrum coromandelianum*, *Cyperus odoratus*, *Fraxinus hookeri* and *Malva parviflora*. Therophytes dominated the life form spectra with 36.36% of plant species, followed by Hemicryptophytes with 25% of species. Nanophyll led the leaf size spectra with 45.45% of species, followed by Microphyll with 22.73% of the species (Table 1). The strongest environmental variables of this IPB community were pH (6.4–6.6), DP (18.6–21.4), and WB (20.8–23.8) and (Supplementary data Figures S1 and S4).

#### 3.2.5. *Sambucus–Cedrus–Desmodium* Community

This plant community (SCD) was recorded with a total of 129 associates in 11 stands between the altitudinal ranges of 1936 to 2373.8 m. *Sambucus weightiana* (5.25 IV), *Cedrus deodara* (5.05 IV), Desmodium elegans (4.1 IV) were recognized as the topmost dominant species. *Sorbaria tomentosa*, *Dactylis glomerata*, *Heracleum candicans*, *Dryopteris wallichiana*, *Pennisetum orientale*, *Onopordum acanthium*, *Fragaria nubicola*, *Foeniculum vulgare*, *Parrotiopsis jacquemontiana* and *Phragmites latissimus* were other co-dominant species with lower IV values as compared to the community indicators. Nonetheless, *Phytolacca latbenia*, *Pleurospermum stellatum*, *P. stylosum*, *Seseli libanotis*, *Torilis japonica*, *Vicia sativa*, *Vincetoxicum petrense*, *Corydalis carinata*, *Polygonatum verticillatum*, *Salix tetrasperma*, *Sida cordata*, *Thalictrum pedunculatum*, *Lindelofia* sp., *Spiraea vaccinifolia* and *Trachyspermum amii* were found as the rare species with less IV values. Therophytic class dominated the life form spectrum, accounting for 43.69% of plant species, followed by nanophanerophytes (19.42%). Nanophyllous leaf size class dominated the SCD community with 31.30% of species, followed by Microphyll (28.24%) and Leptophyll (19.08%). Moreover, one aphyllous plant species was also found in SCD community (Table 1). The most influential ecological variables that influenced the composition of SCD community were K (210–228 mg/kg), P (12.8 mg/kg), pH (5.2–6.9), and EC (3.3 dsm^−1^), and loamy soil texture (Figure 3), which were distributed on a slope angle of 25–85° (Supplementary data Figures S1–S4).

#### 3.2.6. *Indigofera–Cedrus–Pinus* Community

This plant community was recognized in 12 stands on the N-W aspect between altitudinal ranges of 1932.3–2437.8 m, which has 141 associated species (Table 1). *Indigofera heterantha* (10.16 IV), *Cedrus deodara* (5.75 IV) and *Pinus wallichiana* (5.66 IV) were recorded as the leading indicators of this plant community with the highest IV values. Other co-dominant species were *Viburnum grandiflorum*, *Cynodon dactylon*, *Heracleum candicans*, *Bistorta amplexicaulis*, *Poa infirma*, *Isodon rugosus*, *Lathyrus aphaca*, *Prunella vulgaris*, *Plantago major*, *Juglans regia*, *Impatiens brachycentra*, *Pennisetum orientale*, *Euphrasia himalayica* and *Pimpinella stewartii*. Nonetheless, rare species with lower IV values included *Epilobium hirsutum*, *Helianthus annuus*, *Salvia nubicola*, *Epimedium elatum*, *Sonchus asper*, *Avena sativa*, *Conyza japonica*, *Portulaca oleracea*, *Prunus armeniaca*, *Alcea rosea*, *Bauhinia variegata*, *Cotoneaster acuminatus*, *Lotus corniculatus*, *Lindelofia* sp., *Cornus macrophylla* and *Lavatera cachemiriana*. Therophytes dominated the life form spectra accounting 37.59% of all the species in the ICP community, followed by Hemicryptophytes (25.53%), Nanophanerophytes (11.35%), and geophytes (9.22%). The nanophyllous class dominated the leaf size spectra with 31.21% of species, followed by Microphyll (26.95%), and Leptophyll (17.02%). Nonetheless, aphyllous species accounted for the least number of species in the ICP community (1.42% of species, Table 1). The strongest ecological variables that significantly influenced the composition of plant species of ICP community associates were CaCO_3_ (7.5 mg/kg), humidity (49.2–68.5%), and soil texture (silty loam) (Figure 3 and Supplementary data Figures S1–S4).

#### 3.2.7. *Cedrus–Pinus–Parrotiopsis* Community

CPP community was recorded in 17 stands between the altitudinal ranges of 2292–3168 m. The highest number of species (197 species) were recorded in this plant community (Table 1 and Figure 2). *Cedrus deodara* (19.88 IV), *Pinus wallichiana* (17.26 IV) and *Parrotiopsis jacquemontii* (8.5 IV) were recognized as the topmost dominant species. Other co-dominant species with lower IV values than the community indicators included *Cynodon dactylon*, *Oxalis corniculata*, *Clinopodium vulgare*, *Isodon rugosus*, *Indigofera heterantha*, *Impatiens bicolor*, *Fragaria nubicola*, *Geranium wallichianum* and *Clematis grata* were. Moreover, species that were rarely recorded in this community were *Rhynchosia pseudo-cajan*, *Sorbus tomentosa*, *Euphorbia helioscopia*, *Galium asparagifolium*, *Hyoscyamus niger*, *Silene conoidea*, *Poa infirma*, *Rosa webbiana*, *Galium aparine*, *Rumex nepalensis*, *Smilax glaucophylla*, *Spiranthes sinensis* and *Vicia sativa*. Therophytes dominated the life form spectra with 42.31% of species, followed by Hemicryptophytes with 27.47% of species. The microphyllous and Nanophyllous classes dominated the leaf size spectra, accounting for 30.96% of species, followed by Leptophyll (17.77%) and mesophyll (14.72%) (Table 1). Loamy and silty loamy texture, P (11.12 mg/kg), and K (214.6 mg/kg) were all influencing variables for the CPP community (Figure 3 and Supplementary data Figures S1–S4). As a result, the highest species diversity was observed in this CPP community.

#### 3.2.8. *Pinus-Viburnum-Cedrus* Community

The PVC community was recognized on the northern aspect between altitudinal ranges of 2568–3191 m in 7 stands with a total of 195 associated plant species. *Pinus wallichiana-Viburnum grandiflorum-Cedrus deodara* were recorded as the dominant species in this community with highest IV values of 17.98, 16.43, and 9.92, respectively. Other co-dominant and characteristic plant species were *Abies pindrow*, *Arisaema jacquemontii*, *Juniperus squamata*, *Juniperus communis*, *Picea smithiana*, *Fragaria nubicola*, *Cynodon dactylon*, *Quercus incana*, *Urochloa panicoides* and *Bergenia stracheyi*. Moreover, rare species of this PVC community with lower IV values included *Impatiens bicolor*, *Lotus corniculatus*, *Rumex nepalensis*, *Epilobium latifolium*, *Helianthus annuus*, *Inula cuspidata*, *Platanus orientalis*, *Pleurospermum stylosum*, *Pteracanthus urticifolius* and *Swertia paniculata*. Therophytes dominated the life form with 33.66% of species, followed by Hemicryptophytes with 30.69% of species, and Nanophanerophytes with 11.88% of species (Table 1). Nanophyllous dominated the leaf size spectra, accounting for 34.18% of plant species, followed by Microphyll (18.06%) and Leptophyll (16.84%) (Table 1). OM (2.12%), K (215.7 mg/kg), P (11.9 mg/kg), and EC (2.12 dsm^-1^) were the most effective ecological variables that had a positive influence on the species diversity of the PVC community (Supplementary data Figures S1–S4).

#### 3.2.9. *Abies–Picea–Juniperus* Community

This community (APJ) was recorded at the middle altitudinal range (2874–3260 m) of the north-western aspect of the study area with 66 associated plant species (Table 1). The indicators of the APJ community are *Abies pindrow* (26.17 IV), *Picea smithiana* (23.77 IV), and *Juniperus squamata* (21.77 IV). *Thymus linearis*, *Bistorta affinis*, *Bergenia stracheyi*, *Rheum australe*, and *Poa infirma* are some of the herb layer’s co-dominant species, while *Juniperus communis* and *Cotoneaster microphyllus* are the distinguishing species of the shrubby layer. Furthermore, *Quercus incana* and *Pinus wallichiana* are the major tree layer associates with the APJ community. The APJ community is characterised by a preference for the shade. In comparison to sub-alpine (JSJ) and alpine (SBR) communities, hill slopes get less direct sunlight. The shade effect was significantly influenced by the tree layer’s larger canopy cover. Hemicryptophytes dominated the life form classes with 40.98% of species, followed by Therophytes (29.51%), Chamaephytes, and Geophytes (9.84%) each. The nanophyllous class dominated the leaf size spectra, accounting for 33.33% of plant species, followed by Microphyll (27.27%) and Leptophyll (24.24%) (Table 1). Low K (205.4 mg/kg) and low EC (1.4 dsm^−1^) were the most significant ecological variables that played a vital role in the formation of the APJ community. Moreover, the APJ community was hosted by a clay-loamy soil texture (Supplementary data Figures S1–S4) with a low pH (Figure 3).

#### 3.2.10. *Juniperus–Sibbaldia–Juniperus* Community

With a total of 40 associated species, this community (JSJ) was observed in six stands varying in altitude from 3250 to 3644 m (Table 1). The indicators of the JSJ community are shrubs, i.e., *Juniperus squamata*, *Sibbaldia procumbens*, *Juniperus squamata*. The tree layer was represented by the only species (*Rhododendron arboreum*). Other shrubby layer associates of the JSJ community includes *Cotoneaster microphyllus*, and *Juniperus excelsa*, while the herb layer associates were *Bergenia stracheyi*, *Bistorta affinis*, *Caltha palustris*, *Dracocephalum nutans*, *Primula hazarica*, *Poa infirma* and *Rheum australe*. Hemicryptophytes dominated the life form spectra, accounting for 56.82% of plant species, followed by Therophytes (18.18%), Geophytes and Nanophanerophytes (11.36%) each (Table 1). The leaf size spectrum was dominated by Nanophyllous class (34.69% of plant species), followed by Leptophyll (26.53% of plant species), Microphyll (20.41% of plant species) and Mesophyll (14.29% of plant species). Low EC (0.85 dsm^−1^), temperature (7.4–13.8 °C), WS (2–3 m/s) and DP (12.8–15.5) all had a significant impact on the JSJ community. As a result, these ecological variables constrain the species diversity of JSJ community (Supplementary data Figures S1–S4).

#### 3.2.11. *Sibbaldia–Bergenia–Rheum* Community

This community (SBR) was identified at the higher altitudinal ranges (3199–3688 m) above the timber line at latitude (N = 34.69472–34.79333) and longitude (E = 73.60278–73.68639). The SBR community represents subalpine vegetation, having *Sibbaldia procumbens* (10.78 IV), *Bergenia stracheyi* (8.37 IV), and *Rheum australe* (7.75 IV) as the indicator species, for a total of 53 associated species. The herbaceous species dominated the vegetation, although some nanophanerophytes occur at comparatively lower altitudes, i.e., *Juniperus squamata*, *J. communis*, *J. excelsa* and *Cotoneaster microphyllus*. Nevertheless, other herbaceous co-dominants were *Poa alpina*, *P. infirma*, *Bistorta affinis*, and *Primula hazarica*. This subalpine community develops in between the timberline and alpine meadows, regardless of slope aspect, and overlaps with the alpine community (*Poa-Bistorta-Primula*) at most of the elevations. Hemicryptophytes dominated the life form spectra with 48% of species, followed by Therophytes (24% of species) and chamaephytes (12% of species). The Nanophyllous class dominated the leaf size spectra, accounting for 38% of all species, followed by Leptophyll (18%) and microphyll (20%) (Table 1). The altitude and WS (2.5–5 m/s) had a significant impact on this plant community. The indicators, as well as other associated species were also found to be temperature sensitive. The soil texture hosting the SBR community was mainly clay, with the lowest K (197–216.6 mg/kg) and pH (4.8–5.8) values and maximum OM (0.98–2.28%) concentration (Figure 3). Moreover, the SBR community was found to be negatively associated with ecological variables such as humidity, CaCO_3_, HI, WB, BP, and slope angle (Supplementary data Figures S1, S3 and S4).

#### 3.2.12. *Poa-Bistorta-Primula* Community

The *Poa-Bistorta-Primula* Community (PBP) was recognized as the highest altitudinal (3724–4278 m) alpine and cold desert plant community recorded at a latitude (N = 34.69306–34.83861) and a longitude (E = 73.60750–73.69444). *Poa alpina* (17.32 IVI), *Bistorta affinis* (15.03 IV), and *Primula rosea* (9.07 IV) are the indicator species of PBP community. Other co-dominant species are *Rheum australe*, *Bergenia stracheyi* and *Androsace hazarica*. This plant community included a total of 39 species. However, tree and shrub layers (Phanerophytes and Nanophanerophytes) were entirely absent from these alpine meadows. Moreover, this alpine community (PBP) has a low species richness as compared to other plant communities (Figure 2 and Table 1). Extremely low temperatures are a hallmark of the growth period due to high elevation. Xeric conditions compounded such harsh environments, and a relatively short growth season was recorded from July to September. Hemicryptophytes contributed 53.85% of the species, followed by Therophytes (23.08%), Chamaephytes (12.82%), and Geophytes (10.26%). Microphyll and Nanophyll classes dominated the leaf size spectra with 28.95% of species each, followed by Leptophyll (21.05%) (Table 1). Higher altitude and WS (3.5–8 m/s), as well as low temperature had a strong impact on the indicators and other associates of PBP community. The soil texture hosting this community was sandy in nature (Figure 3), with the lowest K (196–206.9 mg/kg) and pH (4.9–5.6) and maximum OM (1.15–2.64%) concentration. Furthermore, the PBP community was found to be negatively associated with ecological variables such as humidity, CaCO_3_, HI, WB, BP, and slope angle (Supplementary data Figures S1–S4).

### 3.3. *Non-Metric Multidimensional Scaling (NMDS)*

**Figure 4:**
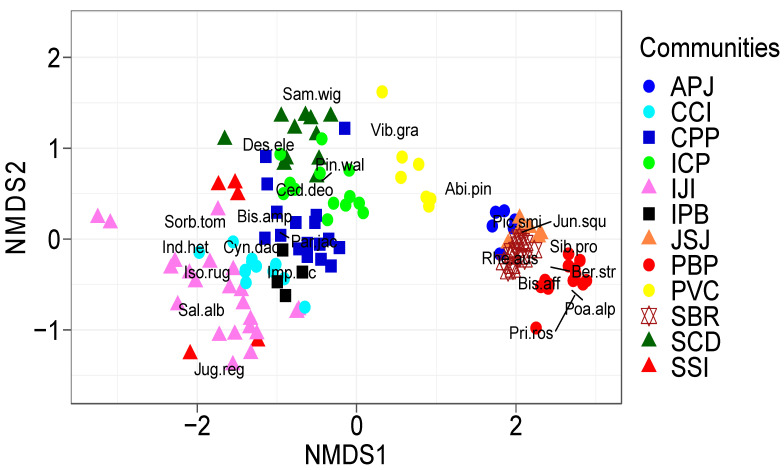
NMDS ordination is based on plant species that are in association with sampled stands and grouped into communities Plant communities that are close together or on the same axis and have a positive correlation. Only the three most representative plant species for each community are plotted. Some species are present in different communities and due to that, the total number of species are not 36.

All the data based on 354 plant species in 133 sampled stands were categorized into 12 plant communities using NMDS. Plant communities that are close together or on the same axis have a positive correlation, whereas communities that are far apart or on different axes have a negative correlation. The *Poa–Bistorta–Primula*, *Sibbaldia–Bergenia–Rheum*, and *Juniperus–Sibbaldia–Juniperus* communities, for example, had a positive association with one another but negatively correlated with the *Indigofera–Juglans–Isodon*, and *Indigofera-Cedrus–Pinus* communities (Figure 4). All these relationships could be attributed to patterns in the variables of their host environment. For example, the former plant communities were found at elevations of 3724–4278 m, 3199–3688 m, and 3250–3644 m, respectively, while the latter plant communities were found at lower elevations (1597–2456 m and 1932.3–2437.8 m), respectively. Moreover, four stands (S38, S39, S40, S41 and S42) are correlated with each other and shaped the SSI community. The PBP community identified in 13 stands can be seen far apart from the other plant communities. Only the three most representative plant species for each community are plotted.

### 3.4. *Canonical Correspondence Analysis (CCA)*

The CCA and variation partitioning tests showed that the total inertia results of CCA was 8.626, where our final variables (Altitude, Slope ES, Slope SE, Slope SW, Slope WN, EC, heat index) together explained 22.6% of variation. CCA model was significant (χ^2^ = 1.951; pseudo-F value = 4.529; *p* < 0.001). For the 8 explanatory variables, we tested simple term effects. Simple term effects showed that all variables were significant (χ^2^_range_ = 0.104–0.785; pseudo-F value_[range]_ = 1.93–14.59; *p* < 0.006; Table 1). Finally, the two first axis were also highly significant (*p* < 0.001). Table 2 displays the significance level of the testing results regarding the influence of environmental variables on the vegetation of the Manoor Valley. The PBP, SBR and JSJ plant communities revealed positive association with altitude (Supplementary data Figures S1–S4). All these plant communities were found at the higher altitudes (3724–4278 m, 3199–3688 m, and 3250–3644 m), respectively. The slope angle, on the other hand, has a negative relationship with altitude. The IJI and ICP plant communities are in positive association with slope angle. This strong influential association might be due to the occurrence of these plant communities in the lower (1597–2456 m) and middle (1932.3–2437.8 m) altitudinal ranges.

## 4. Discussion

Plant species are distributed in diverse types of habitats, forming different communities driven by different sets of environmental variables [6]. The aspect stimulates habitat diversification and promotes micro-environmental variation in the vegetation structure [36,37]. As a result, the composition of different units is observed as a reflection of changing habitat settings along environmental variables [38]. Here, we used different multivariate approaches to assess potential plant communities along an altitudinal gradient and their association with different environmental drivers. Our study documented 354 plant species belonging to 93 families. The current research area is located at the elevations ranging from 1580 m to 4278 m, with varying environmental conditions that are reflected in a rich and diverse flora. Our results showed that at high altitudes, species richness was reduced, whereas an increase in soil nutrients was linked to progression in vegetation indicators. We also found different clusters of species with similar habitats. Our study clearly shows how altitudinal variables can cluster different plant communities according to different microclimates.

In the current vegetational sampling of a remote valley (Manoor Valley, Himalaya), 12 major plant communities were established by TWINSPAN from the lower ranges to the alpine meadows. The CPP communities (197 species) in the middle altitudinal habitats (2292–3168 m) have the most plant species. Ordination methods have commonly been used to show species distribution and community structure along ecological variables [98,99]. Similarly, a researcher investigated the vegetation of the western Himalayas and identified five distinct communities, the most abundant of which were found on north-facing slopes at middle altitudes, where the moisture levels were highest [6]. Thirteen major groups were identified in the vegetation of Kammanassie areas using the TWINSPAN classification [100]. These results, along with ours, show evidence that elevational variables are suitable places to evaluate how changes in environmental variables drive plant community structure and diversity. In addition, these results also show that multivariate approaches are powerful tools for community analysis [43] and can be considered in new ecological studies as statistical methods.

As the study area is in the Himalayan belt, the vegetation was primarily of a Sino-Japanese nature. The plant communities were classified based upon climatic (i.e., temperature, HI, DP, WB, BP, and WS), edaphic (i.e., soil pH, EC, OM, P, K, CaCO_3_, soil texture), and topographic variables (i.e., altitude, altitudinal density, latitude, longitude, slope angle, different exposures, and aspects). The vegetation was classified into different communities/associations [101,102] represented by dominant species based on their importance values [20,103]. At lower elevational ranges (1580.8–2456 m), the plant communities with dominant species were *Salix alba*, *Sorbaria tometosa*, and *Impatiens bicolor* (SSI)*, Indigofera heterantha, Juglans regia*, and *Isodon rugosus* (IJI), *Cedrus deodara, Cynodon dactylon*, and *Isodon rugosus* (CCI), *Indigofera heterantha, Parrotiopsis jacquemontii*, and *Bistorta amplexicaule* (IPB)*,* and *Sambucus weightiana, Cedrus deodara*, and *Desmodium elegans* (SCD) respectively. Similar indicators were recorded by other researchers during a field survey in the Himalayas of Pakistan [104].

The vegetation in the upper altitudinal ranges includes *Pinus wallichiana, Abies pindrow, Indigofera heterantha* and *Viburnum grandiflorum*, which are the representatives of moist temperate forests. These plant species are the temperate zone representatives [30,39,41,105,106]. These plant associations were shaped by the impact of various environmental gradients. Ecosystems respond to numerous simultaneous changes in the environment as these variations differ the diversity and distribution of communities [107,108]. Vegetation in distributions more closely resembles the changes in soil characteristics [109,110,111]. Our results revealed that soil characteristics such as EC, pH, soil texture, OM, K and P had a great impact on plant community distribution and association. Soil variables, altitude, latitude, slope aspect and angle also had a strong influence on species richness, as previously reported by [112].

Dissimilar plant communities were described as those with less than 65% similarity [113,114]. The communities’ similarities were due to shrubs, trees, and perennial plants, while the communities’ dissimilarities were due to Therophytes. The maximum similarity index was noted between SBR and JSJ communities (55.53%), followed by JSJ and APJ communities with 39.71% of similarity. The highest similarity between communities may be due to similar environmental conditions [30], which leads to changes in the species’ habitat. The highest dissimilarity was observed between PBP and IPB communities, JSJ and SSI communities (99.94% each), followed by IJI and PBP communities (99.92%), SSI and PBP communities (99.91%). These results follow the findings of [6,53]. Maximum dissimilarity between communities might be due to wide altitudinal variation among communities [30,115], which represents the presence of different set of species adapted to different set of climatic variables [112,116,117,118].

## 5. Conclusions

To the best of our knowledge, this is the only valley within the Himalayas of Pakistan that has never been explored before, due to its harsh terrain and geographical location. The current study revealed that the sampled area has rich species diversity. The study provides the first ever detailed insights into the spatial distribution and vegetation mapping in response to environmental variables in the study area. The flora of the Manoor Valley consists of 354 plant species belonging to 93 families, distributed into a total of 12 major plant communities, from the lowest altitude to the alpine zones. The *Cedrus–Pinus–Parrotiopsis* community resided at the middle altitudinal ranges (2292–3168 m) was recorded with highest number of associates (197 species). Our study clearly shows how altitudinal variables can cluster different plant communities according to different microclimates, which can be a proxy for future studies evaluating the impacts of climate change on plant communities. Studies such as ours are paramount to better understand how environmental factors influence ecological and evolutionary aspects.

## Figures and Tables

**Table 1:** Percentage of plant species recorded in each plant community according to the biological spectrum (life form) and leaf size.

	Plant Communities											
	SSI	IJI	CCI	IPB	SCD	ICP	CPP	PVC	APJ	JSJ	SBR	PBP
Life form												
Chamaephytes	9.23	10.71	12.68	10.23	10.68	7.09	8.79	5.94	9.84	0.00	12.00	12.82
Geophytes	4.62	5.36	9.86	6.82	7.77	9.22	0.00	11.39	9.84	11.36	8.00	10.26
Hemicryptophytes	15.38	22.32	25.35	25.00	0.00	25.53	27.47	30.69	40.98	56.82	48.00	53.85
Liana	3.08	0.89	2.82	1.14	1.94	0.00	1.65	0.99	0.00	0.00	0.00	0.00
Megaphanerophytes	0.00	3.57	2.82	1.14	5.83	2.13	1.65	2.97	6.56	0.00	0.00	0.00
Mesophanerophytes	13.85	7.14	0.00	3.41	9.71	4.96	2.75	1.98	3.28	0.00	0.00	0.00
Microphanerophytes	4.62	5.36	1.41	3.41	0.97	2.13	0.55	0.50	0.00	2.27	0.00	0.00
Nanophanerophytes	12.31	10.71	9.86	11.36	19.42	11.35	14.84	11.88	0.00	11.36	8.00	0.00
Parasitic	0.00	0.89	1.41	1.14	0.00	0.00	0.00	0.00	0.00	0.00	0.00	0.00
Therophytes	36.92	33.04	33.80	36.36	43.69	37.59	42.31	33.66	29.51	18.18	24.00	23.08
Leaf size												
Aphyllous	1.54	0.89	1.33	1.14	0.76	1.42	0.00	0.00	1.52	2.04	2.00	2.63
Leptophyll	10.77	10.71	10.67	15.91	19.08	17.02	17.77	16.84	24.24	26.53	28.00	21.05
Macrophyllous	1.54	5.36	6.67	1.14	6.11	7.09	5.58	5.61	3.03	2.04	2.00	2.63
Mesophyll	27.69	20.54	10.67	13.64	14.50	16.31	14.72	15.31	10.61	14.29	10.00	15.79
Microphyll	27.69	26.79	30.67	22.73	28.24	26.95	30.96	28.06	27.27	20.41	20.00	28.95
Nanophyll	30.77	35.71	40.00	45.45	31.30	31.21	30.96	34.18	33.33	34.69	38.00	28.95

Plant communities: *Salix–Sorbaria–Impatiens* (SSI), *Indigofera–Juglans–Isodon* (IJI), *Cedrus–Cynodon–Isodon* (CCI), *Indigofera–Parrotiopsis–Bistorta* (IPB), *Sambucus–Cedrus–Desmodium* (SCD), *Indigofera–Cedrus–Pinus* (ICP), *Cedrus–Pinus–Parrotiopsis* (CPP), *Pinus–Viburnum–Cedrus* (PVC), *Abies–Picea–Juniperus* (APJ), *Juniperus–Sibbaldia–Juniperus* (JSJ), *Sibbaldia–Bergenia–Rheum* (SBR), *Poa–Bistorta–Primula* (PBP).

**Table 2:** Detailed numerical results of variations partitioning (partial CCA) for groups of variables.

Gradient Class	Variation (adj)	% of Explained	Eigen Values				F	*p*
			Axis 1	Axis 2	Axis 3	Axis 4		
Edaphic	9.8	18.7	0.6211	0.1887	0.1135	0.0904	2.1	0.002
Climatic	9.8	13.9	0.7210	0.1138	0.0841	0.0546	3.4	0.002
Physio-graphic	18.1	25.5	0.7641	0.2903	0.2176	0.1624	3.4	0.002
Aspect	9.1	15.3	0.4280	0.1675	0.1417	0.1206	2.5	0.002

## Supplementary Materials

The following supporting information can be downloaded at: https://www.mdpi.com/article/10.3390/plants11010087/s1. Figure S1: Canonical correspondence analysis: (a) & (c) the contour plot shows the count of species at each axis, (b) Distribution of plant species along the edaphic variables, (d). Distribution of plant species along the climatic variables. Figure S2: Canonical correspondence analysis: (a). the contour plot shows the count of species at each axis, (b). Distribution of plant species along the slope aspects. Figure S3: Canonical correspondence analysis: (a) Distribution of stands along the edaphic variables, (b) and (c) Van Dobben circles show the correlation of species in association to edaphic variables and among them, i.e., red circle shows the positive and blue indicates the negative correlation. Figure S4: Canonical correspondence analysis: (a) Association of sampling sites along the climatic variables, (b). Association of sampling sites along the slope aspects.
