# Supplementary material for: Classification and Characterization of the Manoor Valley’s (Lesser Himalaya) Vegetation from the Subtropical-Temperate Ecotonal Forests to the Alpine Pastures along Ecological Variables
Source: Plants (Basel). 2021 Dec 28;11(1):87. doi: 10.3390/plants11010087 (PMC8747448; doi:10.3390/plants11010087)
Supplement: Supplementary file 1 [file plants-11-00087-s001.zip › plants-1433609-supplementary.pdf]

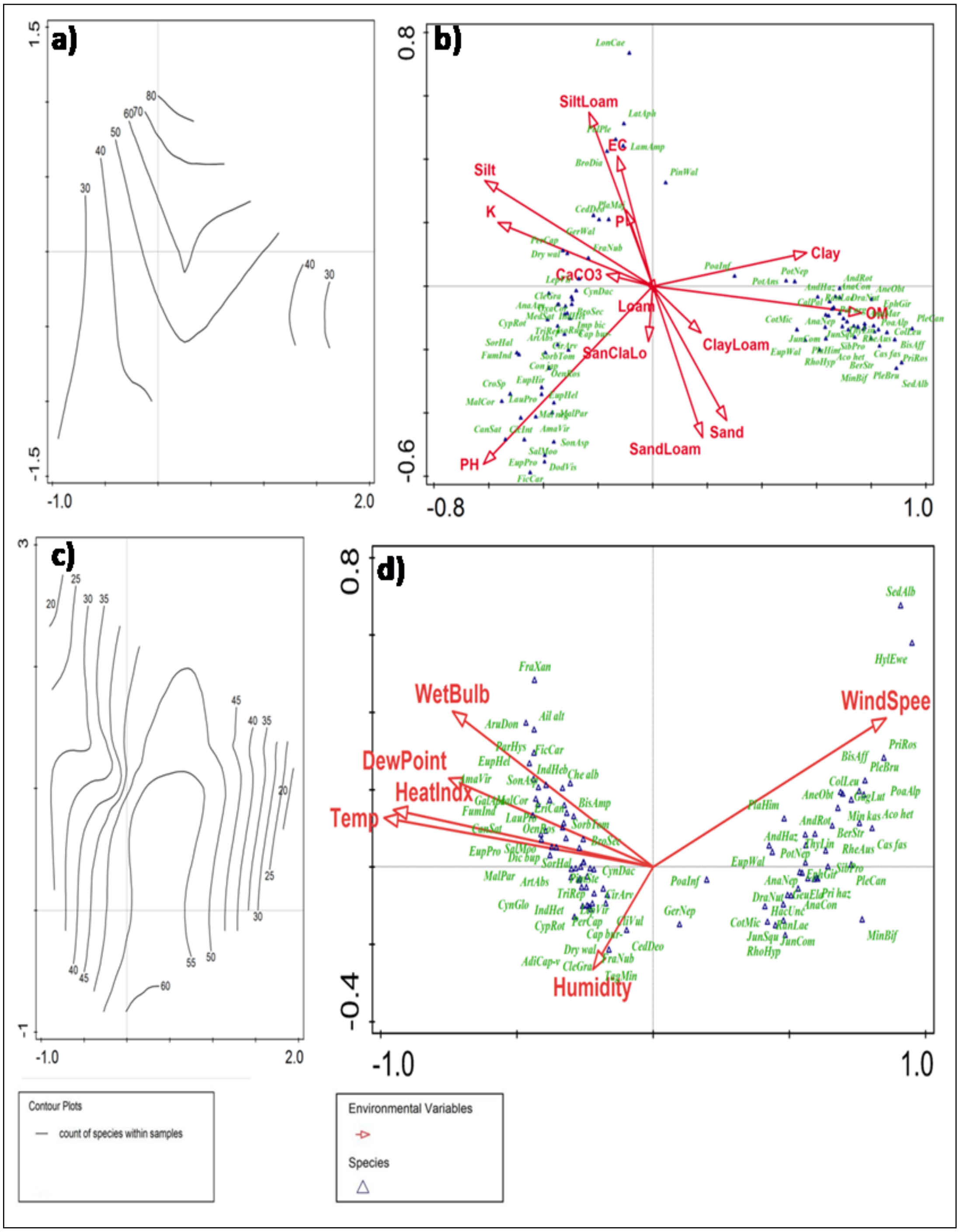

Figure S1: Canonical correspondence analysis: a) & c) the contour plot shows the count of species at each axis, b) Distribution of plant species along the edaphic variables, d). Distribution of plant species along the climatic variables.

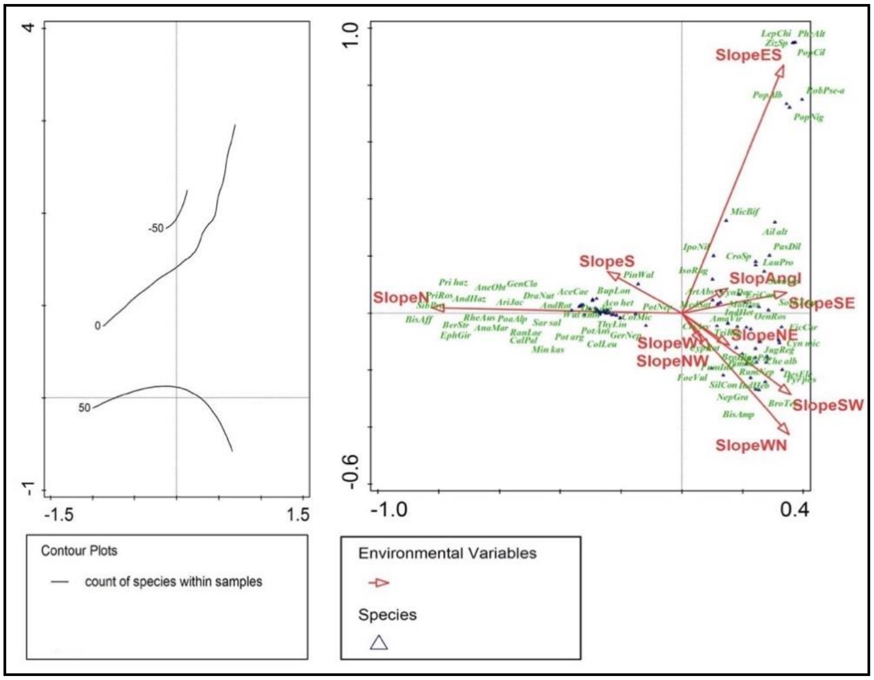

Figure S2: Canonical correspondence analysis: a). the contour plot shows the count of species at each axis, b). Distribution and association of plant species along the slope aspects.

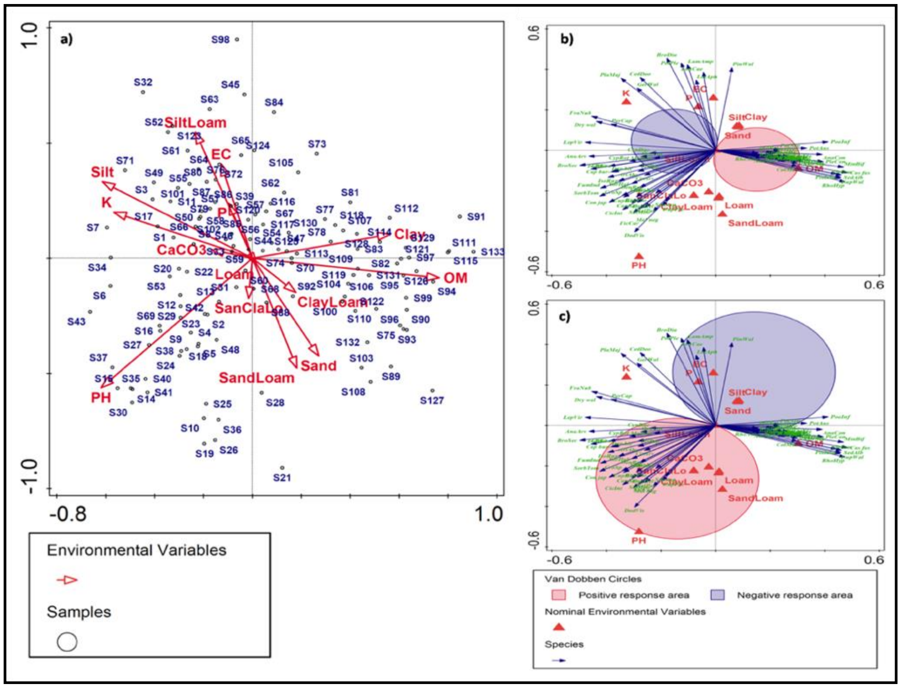

Figure S3: Canonical correspondence analysis: a) Distribution of stands along the edaphic variables, b) and c) Van Dobben circles show the correlation of species in relation to edaphic variables and among them i.e., red circle shows the positive and blue indicates the negative correlation.

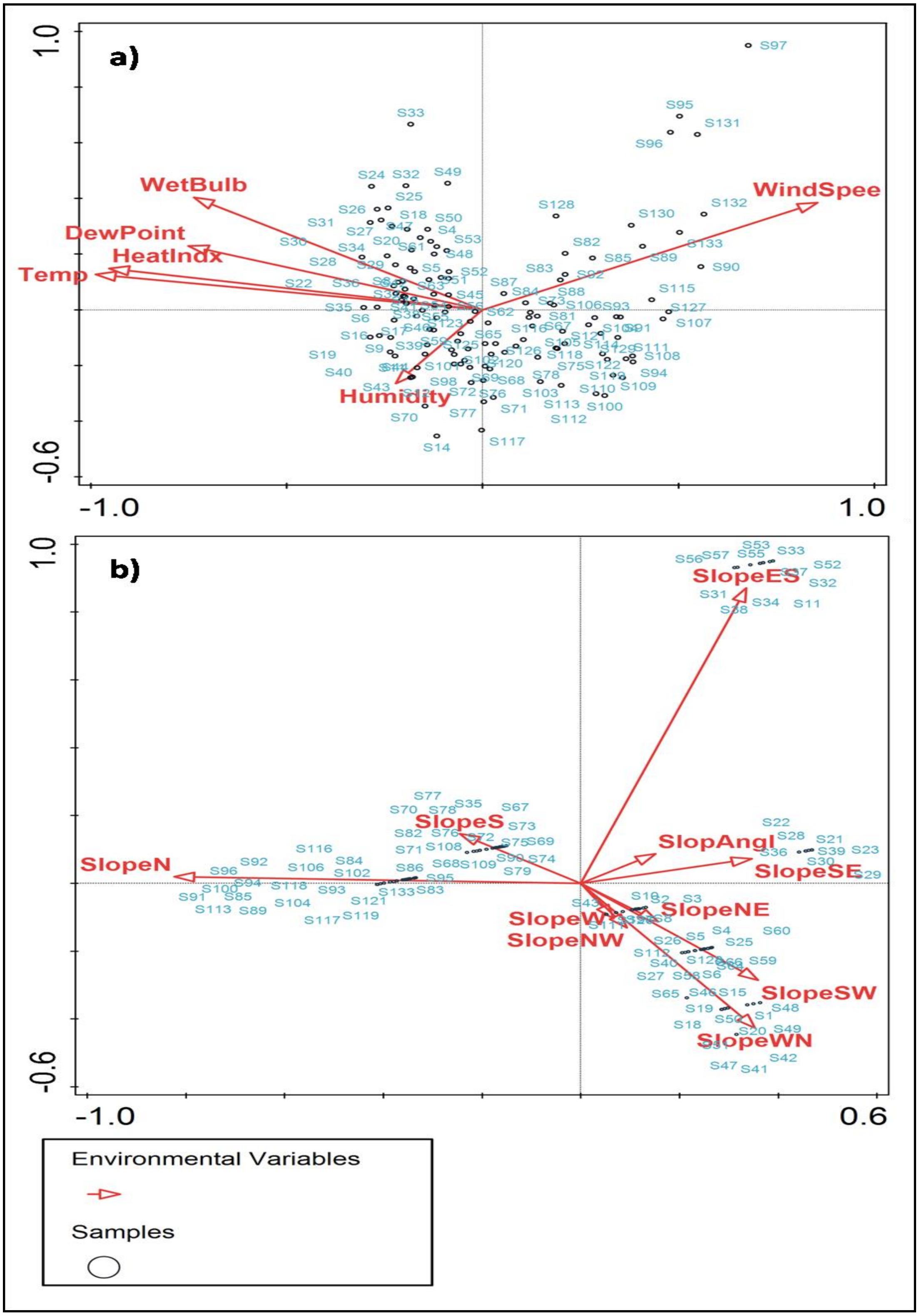

Figure S4: Canonical correspondence analysis: a) Association of sampling sites along the climatic variables, b). Association of sampling sites along the slope aspects.
